# The underlying molecular mechanisms and prognostic factors of RNA binding protein in colorectal cancer: a study based on multiple online databases

**DOI:** 10.1186/s12935-021-02031-6

**Published:** 2021-06-30

**Authors:** Qinglian He, Ziqi Li, Xue Lei, Qian Zou, Haibing Yu, Yuanlin Ding, Guangxian Xu, Wei Zhu

**Affiliations:** 1 Department of Pathology, Guangdong Medical University, No.1 Xincheng Road, Dongguan, 523808 Guangdong Province China; 2 School of Public Health, Guangdong Medical University, Dongguan, 523808 Guangdong Province China; 3 Guangdong Provincial Key Laboratory of Medical Molecular Diagnostics, School of Medical Technology, Institute of Clinical Laboratory, Guangdong Medical University, Dongguan, 523808 Guangdong Province China

**Keywords:** RNA binding protein, Alternative splicing, Colorectal cancer

## Abstract

**Background:**

RNA binding protein (RBP) is an active factor involved in the occurrence and development of colorectal cancer (CRC). Therefore, the potential mechanism of RBP in CRC needs to be clarified by dry-lab analyses or wet-lab experiments.

**Methods:**

The differential RBP gene obtained from the GEPIA 2 (Gene Expression Profiling Interactive Analysis 2) were performed functional enrichment analysis. Then, the alternative splicing (AS) events related to survival were acquired by univariate regression analysis, and the correlation between RBP and AS was analyzed by R software. The online databases were conducted to analyze the mutation and methylation of RBPs in CRC. Moreover, 5 key RBP signatures were obtained through univariate and multivariate Cox regression analysis and established as RBP prognosis model. Subsequently, the above model was verified through another randomized group of TCGA CRC cohorts. Finally, multiple online databases and qRT-PCR analysis were carried to further confirm the expression of the above 5 RBP signatures in CRC.

**Results:**

Through a comprehensive bioinformatics analysis, it was revealed that RBPs had genetic and epigenetic changes in CRC. We obtained 300 differentially expressed RBPs in CRC samples. The functional analysis suggested that they mainly participated in spliceosome. Then, a regulatory network for RBP was established to participate in AS and DDX39B was detected to act as a potentially essential factor in the regulation of AS in CRC. Our analysis discovered that 11 differentially expressed RBPs with a mutation frequency higher than 5%. Furthermore, we found that 10 differentially expressed RBPs had methylation sites related to the prognosis of CRC, and a prognostic model was constructed by the 5 RBP signatures. In another randomized group of TCGA CRC cohorts, the prognostic performance of the 5 RBP signatures was verified.

**Conclusion:**

The potential mechanisms that regulate the aberrant expression of RBPs in the development of CRC was explored, a network that regulated AS was established, and the RBP-related prognosis model was constructed and verified, which could improve the individualized prognosis prediction of CRC.

**Supplementary Information:**

The online version contains supplementary material available at 10.1186/s12935-021-02031-6.

## Background

Colorectal cancer (CRC) is one of the most fatal primary digestive tract tumors [1, 2]. Despite some improvements in diagnosis and treatment, global mortality remains high [1]. At present, the field of CRC research is focused on the development of tools for early detection, reliable prognosis and predictive biomarkers, as well as new treatments that can overcome drug resistance [3–5]. Gene regulation in eukaryotes is a multi-step process and new RNA formed after transcription is usually modified, transported, localized and translated. With the emergence of high-throughput technology in genomics and the new viewpoint of genetic and epigenetic mechanisms, the research has been concentrated on the change of transcriptional level [6]. Many studies often showed that there is a lack of significant correlation between transcripts and protein levels in cells [7]. These observations lead the public to believe that other processes may also play an important role in the cell pool that affects the translation of proteins from their respective transcripts. This paradox can be further explained by identifying post-transcriptional regulatory points, which make a great contribution to the regulation of protein level. These checkpoints are mainly composed of regulation mediated by non-coding RNAs (microRNAs, circular RNAs and long non-coding RNAs) and RNA-binding proteins (RBPs) [8, 9].

RBPs bind to newborn RNAs in the whole process of cells [10]. The versatility and wide range of RBPs targets make them critical post-transcriptional regulatory factors [10]. Therefore, it is necessary to understand the structure and function of these molecules for comprehending many processes that have changed due to the dyregulation of these proteins. Many of these dysregulated RBPs have also been shown to contribute to the pathogenesis of cancer [11].

In this study, besides exploring the potential mechanism of regulating the abnormal expression of RBPs, it was also found that using multiple RBP integrated model, RBP may affect the prognosis of CRC, thus improving the prediction accuracy of prognosis. We procured the results of functional analysis of differentially expressed RBPs, in CRC from online database, which prompted the construction of an alternative splicing (AS) network of CRC after acquiring differentially expressed AS events related to the prognosis of RBP. We hypothesized that gene mutation and DNA methylation were the potential mechanisms for regulating aberrant expression of RBP. Hence, a number of online databases were used to analyze and verify that mutation and DNA methylation were involved in the regulation of aberrant expression of RBPs. Moreover, univariate and multivariate proportional hazard regression analysis were applied to further screen prognostic RBP genes from The Cancer Genome Atlas (TCGA) CRC cohorts and establish the optimal risk model, which was verified in randomized test group. Finally, through the analysis of various publicly available data sets, the expressions of the model's RBPs in CRC were further analyzed.

## Materials and methods

### Acquisition of RBPs

Based on the data reported by Gerstberger et al. in 2014 [12], a complete list of 1542 RBPs used in this study was obtained (Additional file 1). This list was used for all the analyses in this study.

### Differential expression analysis and functional enrichment analysis

The abnormally expressed genes in CRC samples were collected on the Gene Expression Profiling Interactive Analysis 2 (GEPIA 2) database (http://gepia2.cancer-pku.cn/#index) (condition set to Dataset: COAD or READ, |Log2FC| Cutoff: 1; q-value Cutoff: 0.05; Differential Methods: LIMMA), of which 300 RBP genes were differentially expressed in CRC (Additional file 2). Using R software, the gene name was converted to entrezID by referring to "org.Hs.eg.db" package. Then the R packets "clusterProfiler", "org.Hs.eg.db", "enrichplot" and "ggplot2" were performed to analyze the enrichment of gene ontology (GO) and Kyoto Encyclopedia of Genes and Genomes (KEGG) and to visualize the results.

### AS data analysis

The transcriptome and clinical information of CRC were obtained from the TCGA GDC platform (https://portal.gdc.cancer.gov/; released before October 27, 2019), and the AS data of TCGA CRC samples were taken from the TCGA SpliceSeq platform (https://bioinformatics.mdanderson.org/TCGASpliceSeq/PSIdownload.jsp; released before July, 10, 2020). Univariate Cox analysis with R software was used to find AS events related to survival (Additional file 3). The correlation (correlation coefficient R > 0.55, P value < 0.001) between RBP expression and the Percent-spliced-in (PSI) value of survival-related AS was analyzed using the function cor.test () in R software. The network diagram was generated by Cytoscape (version 3.7.1).

### Mutation analysis and methylation data analysis of RBP gene

The gene mutation data of 8930 CRC samples were downloaded from COSMIC website (https://cancer.sanger.ac.uk/cosmic) (Additional file 4), and the gene mutation frequencies of 1542 RBP were investigated. The mutation of RBP with high mutation frequency in CRC was further studied graphically in cBioPortal (http://www.cbioportal.org/). The methylation data of 8930 CRC samples were obtained on the Catalogue of Somatic Mutations in Cancer (COSMIC) website (Additional file 5) to investigate the methylation of abnormally expressed RBP genes in CRC. In addition, we used the TCGA 450 k array downloaded from UCSC (https://xenabrowser.net/datapages/; released before July, 20, 2020) (Additional file 5) to analyze the independent prognosis of TCGA CRC methylation sites and found that there were prognostic methylation sites in RBP. Finally, the data of the expression level and the corresponding methylation degree of the interested RBP genes were retrieved on Cancer Cell Line Encyclopedia (CCLE) (https://portals.broadinstitute.org/ccle) (Additional file 6), and the Pearson analysis was carried out by using Graphpad 8.0 software.

### Construction and verification of prognostic model

Genes related to CRC survival were taken from the GEPIA 2 database (condition setting: Dataset selection: COAD or READ, Methods: overall or disease free survival; Group Cutoff: Median), of which 96 RBP genes were related to CRC survival (Additional file 2). The transcripts and clinical information of 96 RBPs mentioned above were downloaded from the TCGA GDC platform (https://portal.gdc.cancer.gov/; released before October 27, 2019), and the survival-related RBP with significant difference was identified by univariate regression analysis using the 'survival' package of R software. Using R software and data partition function createDataPartition (), the patients with TCGA CRC were randomly divided into train group and test group (Additional file 7). Then multivariate regression analysis was carried out according to the data of train group and the prognosis model was constructed.

In order to verify the accuracy of the model, the calculation formula of the constructed model was first used: the risk score of the sample = ∑ (the Cox regression coefficient X RBP expressed by the log2 ratio value of a specific RBP). The risk score of the train group was calculated, and the risk was divided into two groups according to the median value of the risk. After that, R software was used for Kaplan–Meier (KM) survival analysis, univariate and multivariate independent prognostic analysis, and the receiver operator characteristic (ROC) curve drawing. Finally, according to the median value of train group, test group was also divided into high- and low-risk groups. KM survival analysis, univariate and multivariate independent prognostic analysis and the ROC curve were also carried out.

### The five RBP signatures of the model are validated at transcription and protein levels

The expression data of five RBP signatures in various CRC cell lines were obtained from the CCLE platform (Additional file 8), and their expressions in cell lines from different sources were analyzed. The expression of the five RBP signatures at the CRC organizational level was searched on The Human Protein Atlas (https://www.proteinatlas.org/).

### Cell lines, RNA isolation and qRT-PCR

CRC cells (HCT 116, SW480, SW620, Caco2, RKO) and normal intestinal epithelial cells (NCM460) grew in 1640 (GIBCO; Thermo Fisher Scientific, Inc.) supplemented with 10% FBS (Sera Gold, Germany), and 1% penicillin/streptomycin. RNA was isolated using TRI reagent (SIGM-Aldrich, USA) following manufacturer's instructions. Synthesis of cDNA was using the Prime-Script RT reagent kit (Takara Bio, Inc.), 2.0 μg RNA was converted into cDNA. qRT-PCR was performed to quantify the transcript levels under various conditions. GAPDH was used as an internal control, and the ΔΔCt method was used to calculate gene expression. The primers used in this study were CAPRIN2 (forward): 5’-CAGAGACTCCTGAGGCAGCAATTC-3’, CAPRIN2 (reverse): 5’-GAAGCCCTGTTCAGAGCCCTTTG-3’, GAPDH (forward): 5’-CTCCTCCTGTTCGACAGTCAGC-3’, GAPDH (reverse): 5’-CCCAATACGACCAAATCCGTT-3’. Statistical analysis was performed using GraphPad Prism version 8.0 (GraphPad Software, Inc.). The data were expressed as mean ± standard deviation. The student’s t test was used to compare the two groups. P value < 0.05 was considered to indicate a statistically significant difference.

## Results

### Differentially expressed RBPs in CRC and their enrichment network

**Fig. 1:**
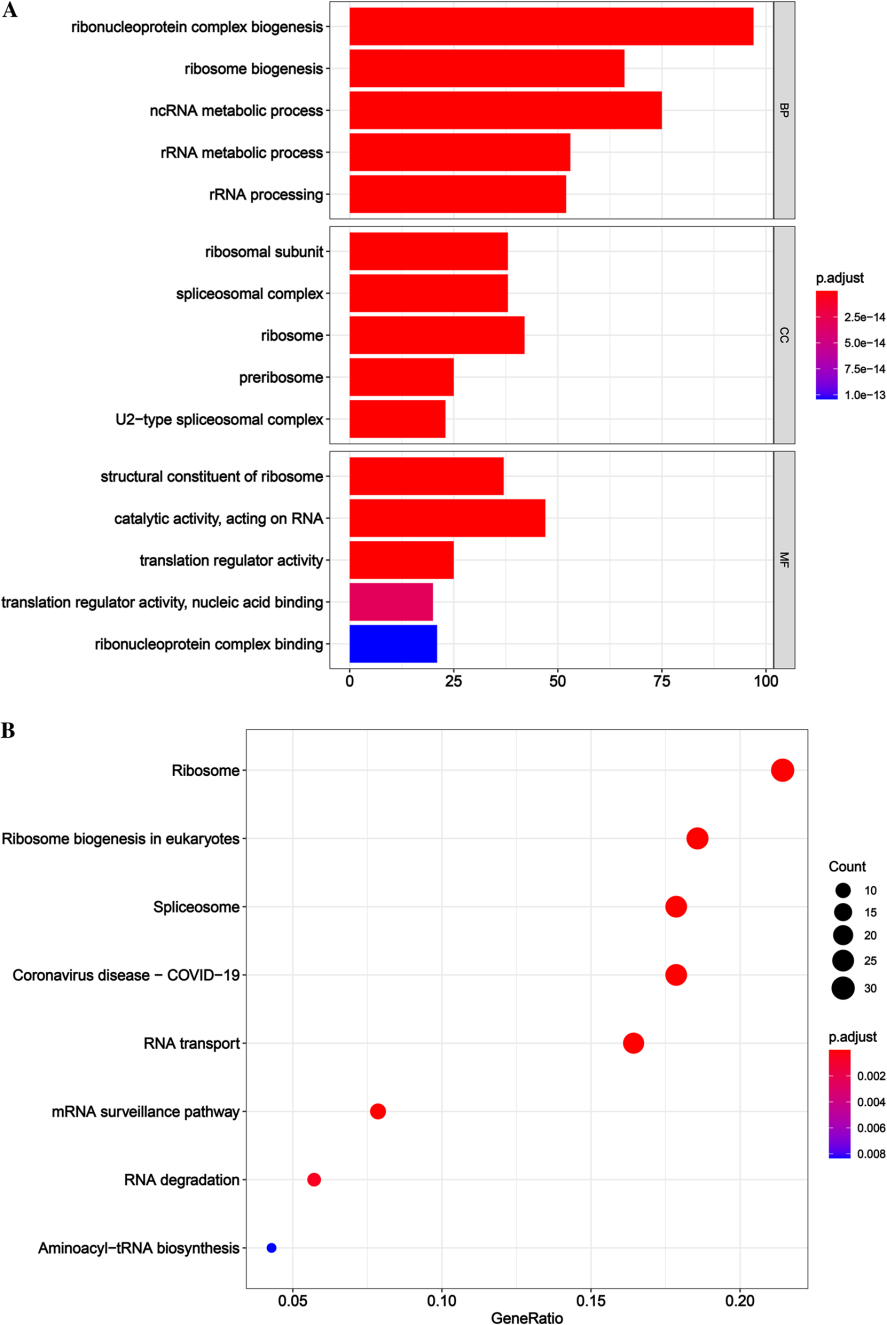
Functional enrichment analysis of RBP gene abnormally expressed in CRC. **A** is the results of biological process (BP), cellular component (CC) and molecular function (MF) enrichment in GO analysis of abnormally expressed RBP genes in CRC. **B** is the result of enriched signal pathway in KEGG analysis

We acquired an exhaustive list of 1542 RBPs from the literature published by Gerstberger et al. (Additional file 1). The CRC differential genes were downloaded from the GEPIA 2 online database, and it was found that there were 71 down-regulated RBP genes and 229 up-regulated RBP genes in CRC (Additional file 2). In order to understand the potential mechanism of RBP in CRC, KEGG analysis and GO analysis were performed on the above 300 differentially expressed RBP genes (Fig. 1).

### RBPs in CRC participate in the network regulation of AS

**Fig. 2:**
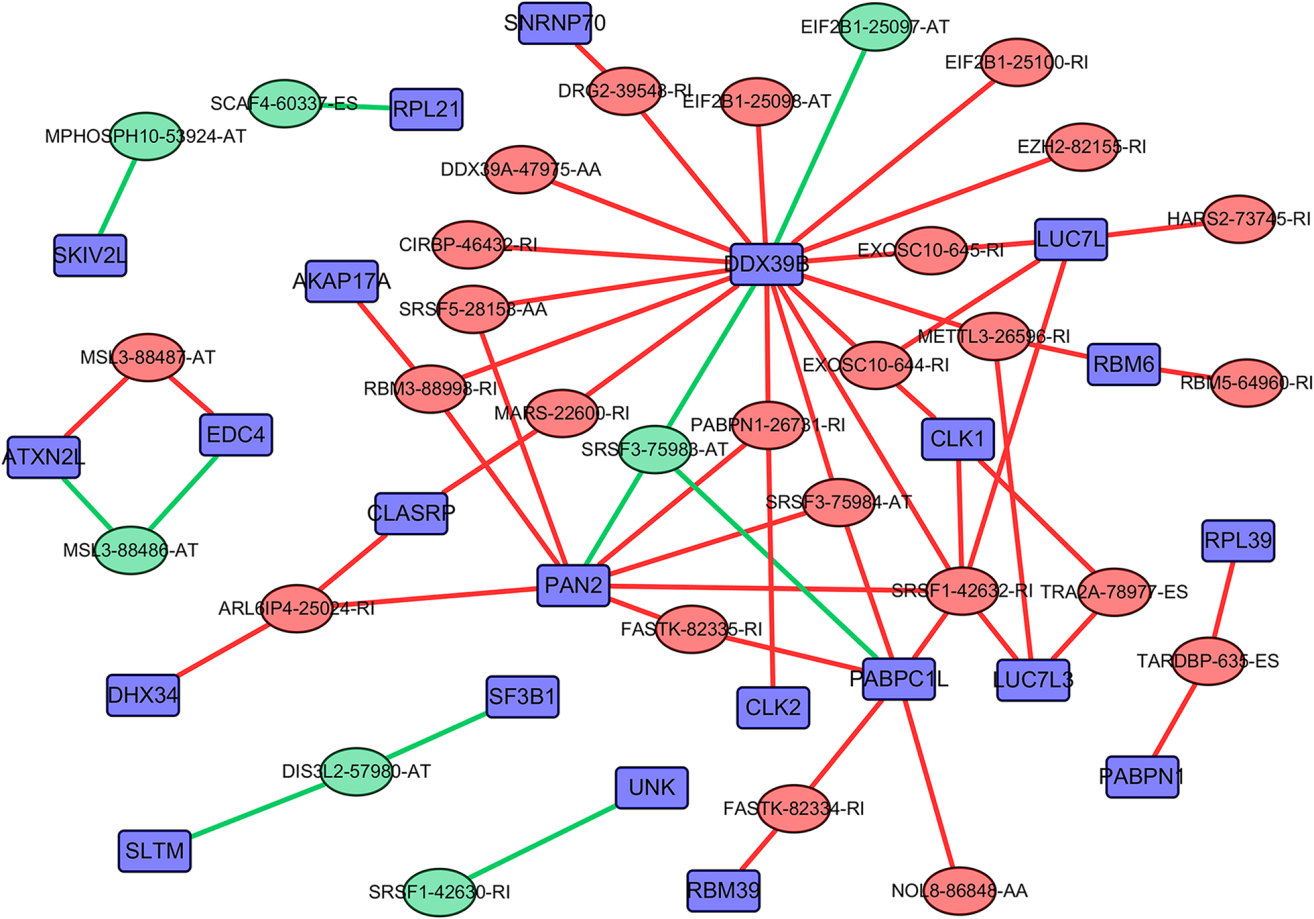
Network diagram of RBPs regulating AS events. The purple boxes represent the RBPs, red ellipses represent the up-regulated AS events in CRC, and the green ellipses represent the down-regulated AS events in CRC. The red lines indicate that RBPs positively regulated AS events, while the green lines indicate that RBPs negatively regulated AS events

As shown in Fig. 1 that RBPs were enriched in the spliceosome pathway, indicating that RBPs were important part of participating in AS. As we all know, AS events are mainly regulated by splicing factors. Splicing factors bind to pre-mRNA and affect exon selection and splicing site selection [13]. More importantly, AS disorders in the tumor microenvironment may be caused by a limited number of splicing factors [14]. Thus, an important question was whether a large part of these AS events related to the prognosis of CRC (Additional file 3) may be regulated by some key splicing factors. According to the data collected by zhen et al. in 2018 [15], most of the splicing factors belong to RBPs. Therefore, the regulatory relationship between RBPs and AS events was worth exploring. For this purpose, the expression of RBPs from the RNA sequencing data of the TCGA CRC cohort was analyzed. Next, in the CRC cohort, through the splicing regulation network established by significant correlation (|R|> 0.55, t test, *P* < 0.05), the correlation between the expression levels of these 1542 RBPs and the PSI value of each AS events related to the prognosis of CRC was analyzed. In the splicing-related network shown in Fig. 2, there were 31 AS events related to the prognosis including 24 up-regulated AS events (red dots) and 7 down-regulated AS events (green dots) significantly correlated with 22 RBPs (purple dots). Interestingly, most RBPs (purple dots) were associated with more than one AS event, some of which played an opposite role in the regulation of differential AS events. Moreover, our network revealed that different splicing factors competed for the same binding site (AS event), which at least partly explained why the transcript was able to produce several different splicing isoforms. In addition, we observed from the Fig. 2 that DDX39B as RBP had the most AS events, mainly up-regulating AS events. This showed that DDX39B may be a key factor in modulating AS events related to CRC prognosis.

### Potential mechanisms that may be involved in the regulation of differentially expressed RBP genes in CRC

**Fig. 3:**
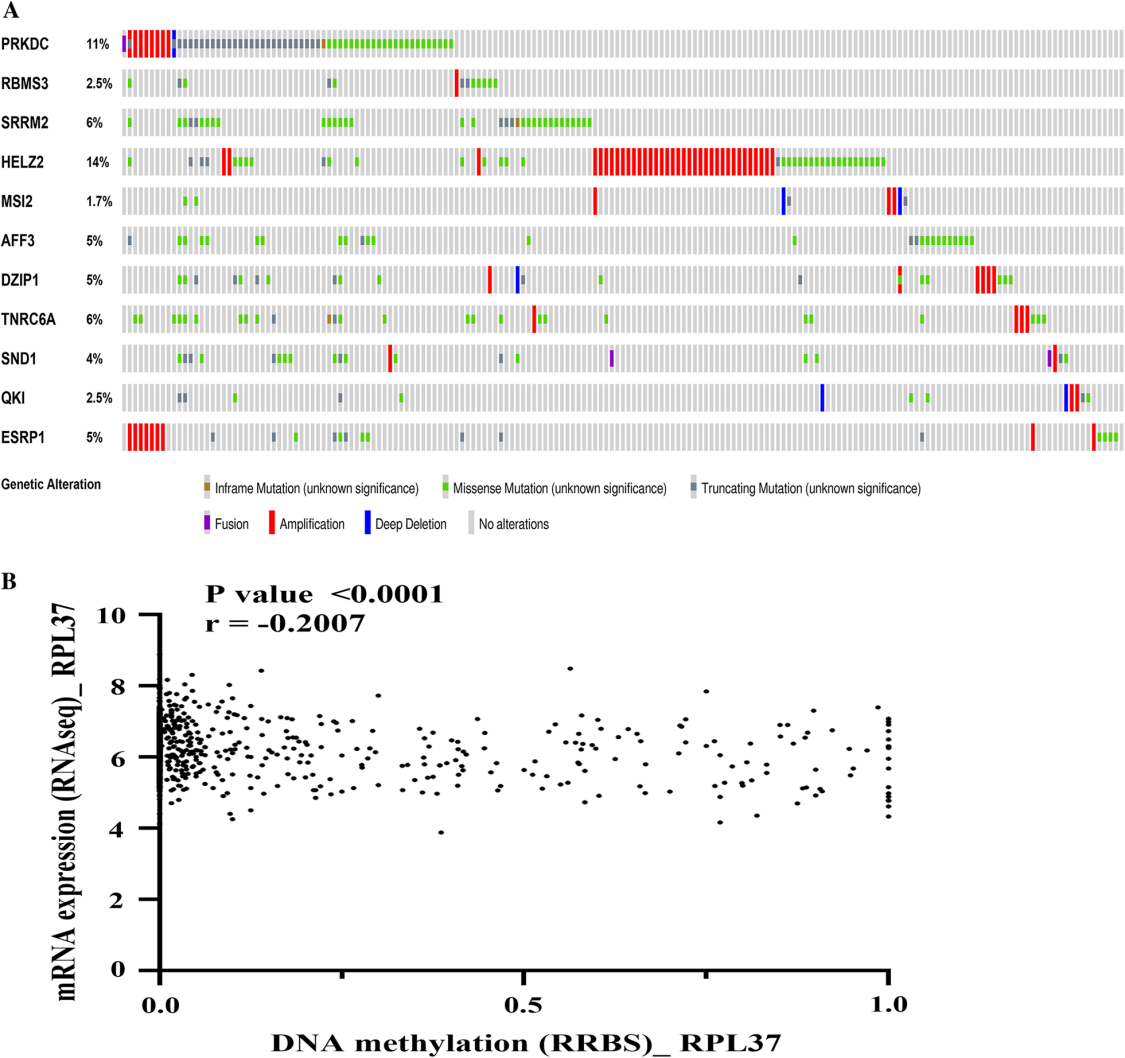
**A** Mutations of 11 interest genes in CRC. The graph shows CRC samples with gene mutation. Each gray bar represents a CRC sample. The brown stripes denote samples with inframe mutations in particular RBPs, the green stripes indicate the samples with missense mutation, while the black stripes indicate that truncating mutations were identified in RBP of CRC samples. **B** Analysis of the correlation between methylation degree and expression of RPL37 in CRC cell lines

Gene mutations are ubiquitous and occur spontaneously. Not all mutations cause obvious changes in cell functions [16]. However, mutations in key cellular genes cause developmental disorders, which is one of the main ways that proto-oncogenes are transformed into a carcinogenic state [17]. The gradual accumulation of multiple mutations in life will result in cancer, which is also one of the important mechanisms for the occurrence and development of CRC [18]. We speculated that the underlying mechanism of RBP gene differential expression may be caused by mutations, so we used 8,920 CRC samples on the COSMIC platform to investigate the occurrence of differentially expressed RBP gene mutations. We were surprised to discover that all the differentially expressed RBP genes were mutated in CRC samples, and the mutation frequency of 11 RBP genes (PRKDC, RBMS3, SRRM2, HELZ2, MSI2, AFF3, DZIP1, TNRC6A, SND1, QKI, ESRP1) was more than 5% (Additional file 4). Then, in order to further understand the mutations of the first 11 RBP genes, we analyzed 526 TCGA colorectal adenocarcinoma samples (TCGA, pancancer Atlas) on the cBioportal platform, and 180 (34%) samples showed mutations (Fig. 3A). The mutation frequency of these 11 differentially expressed RBP genes in CRC was indeed high, and the mutation frequency of PRKDC and HELZ2 was more than 10% in the Fig. 3A. These two genes may be key therapeutic targets for CRC.

Additionally, we also noticed the role of epigenetics in CRC. Therefore, we investigated the methylation of differentially expressed RBP genes on 8920 CRC samples on the COSMIC platform and revealed that 21 differentially expressed RBP genes had DNA methylation changes (Additional file 5). Thus, we used the TCGA 450 k array to perform independent prognostic analysis of TCGA CRC methylation sites and determined that 64 RBPs with prognostic methylation sites (Additional file 5) included 10 differentially expressed RBP genes (RPL37, NOL10, CD3EAP, EIF5A, OASL, NHP2, RRS1, NUFIP1, RRP12 and EIF4E). Then we tested the correlation between the degree of methylation of these 10 genes on CRC cell lines and their expression on the CCLE platform. It was observed that the degree of RPL37 methylation was negatively correlated with its mRNA expression level (Fig. 3B).

### Construction of RBPs related prognosis model

**Fig. 4:**
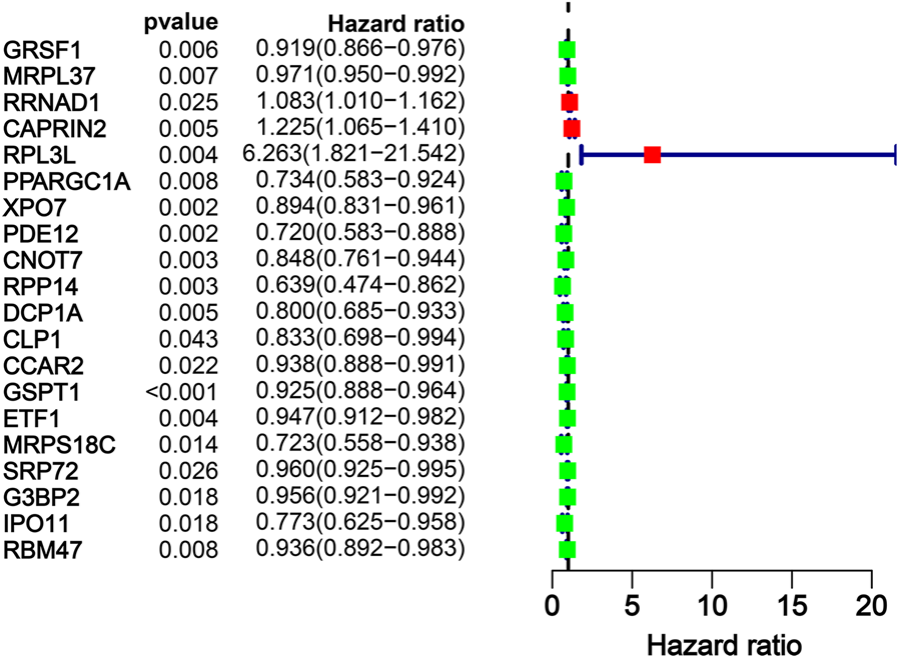
Multivariate regression analysis identified RBP genes associated with CRC prognosis

RBPs play an important role in the occurrence and development of CRC [19]. We collected 1,855 survival-related genes for CRC through the GEPIA 2 database and found that among the 1542 RBPs that have been cataloged, only 96 RBPs were related to the survival of CRC. Simultaneously, CRC transcripts and clinical information from the TCGA platform were downloaded, and data integration was carried out to obtain the clinical information of 540 CRC patients using R software. Thus, the 96 RBPs obtained above were subjected to univariate cox regression analysis to further screen twenty RBPs related to CRC survival (Fig. 4). Next, using R software, the above 540 CRC patients were randomly divided into two groups according to their survival status (to ensure that the number of surviving patients and the number of dead patients is not much different), namely the train group and the test group (Additional file 4). Finally, according to the train group, a multivariate analysis was performed to construct a CRC prognostic prediction model of five genes (CAPRIN2, RPL3L, CCAR2, GSPT1 and MRPS18C) (Table 1).

**Table 1:** Five RBP signatures for constructing prognosis model

Gene symbol	Full name	Functions	Coef	HR
CAPRIN2	Caprin family member 2	Regulation of mRNA transport Differentiation Growth regulation	0.194159638	1.214290114
RPL3L	Ribosomal protein L3 like	Ribonucleoprotein Ribosomal protein	2.641957965	14.04066785
CCAR2	Cell cycle and apoptosis regulator 2	Transcription regulation Wnt signaling pathway Tumor suppressor	− 0.061645845	0.94021581
GSPT1	G1 to S phase transition 1	Nonsense-mediated mRNA decay Protein biosynthesis	− 0.065819488	0.936299863
MRPS18C	Mitochondrial ribosomal protein S18C	Ribonucleoprotein Ribosomal protein	− 0.454631634	0.63468172

### Validation of RBPs related prognostic model

**Fig. 5:**
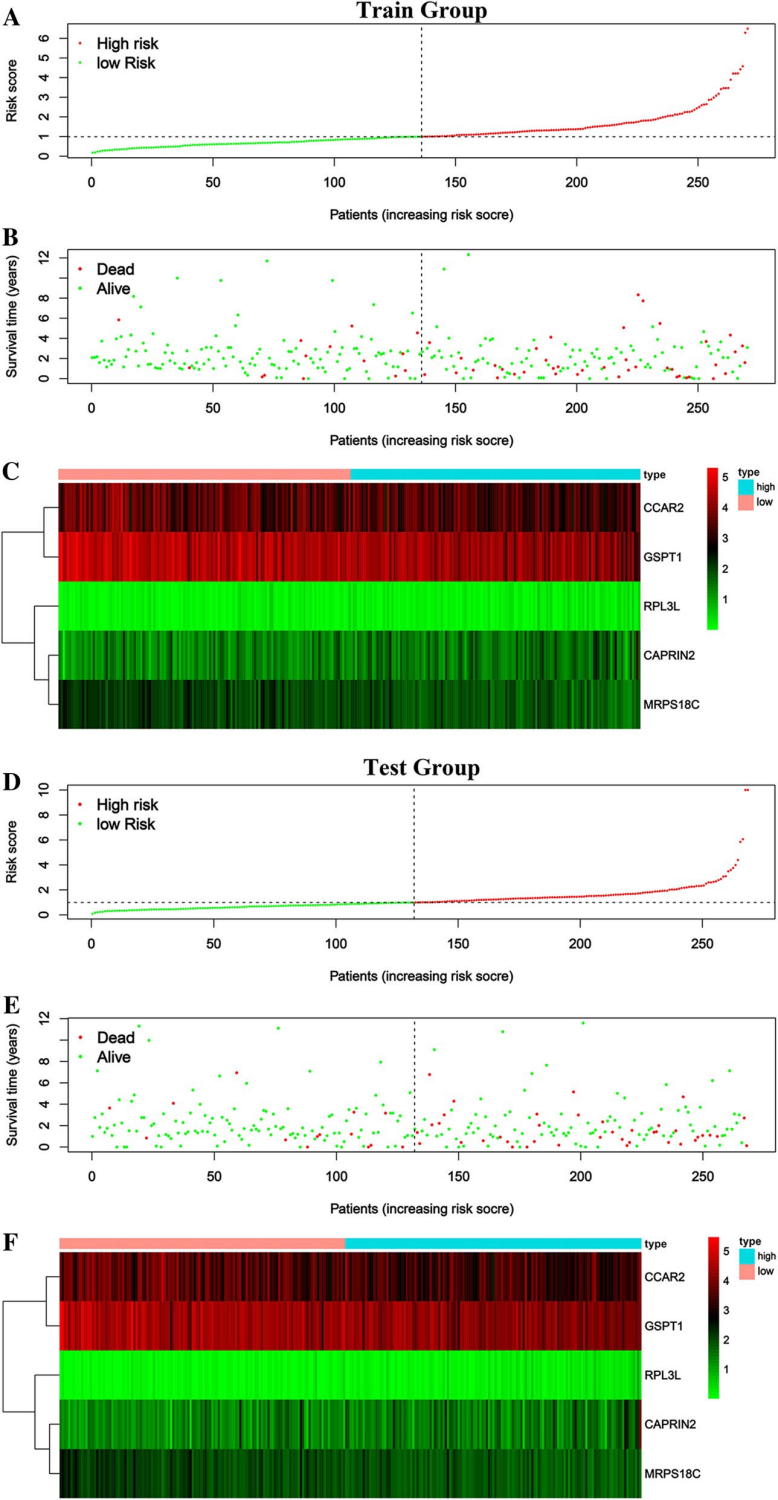
Characteristics of the prognostic gene signatures. The distribution of risk score and patient’s survival time, as well as status for Train group (**A**–**B**) and Test group (**D**–**E**). (**A**–**B** and **D**–**E**) The black dotted line is the optimum cutoff dividing patients into low-risk and high-risk groups. (**C** and **F**) Heatmap of the RBP gene expression profiles in prognostic signature for Train group (**C**) and Test group (**F**)

**Fig. 6:**
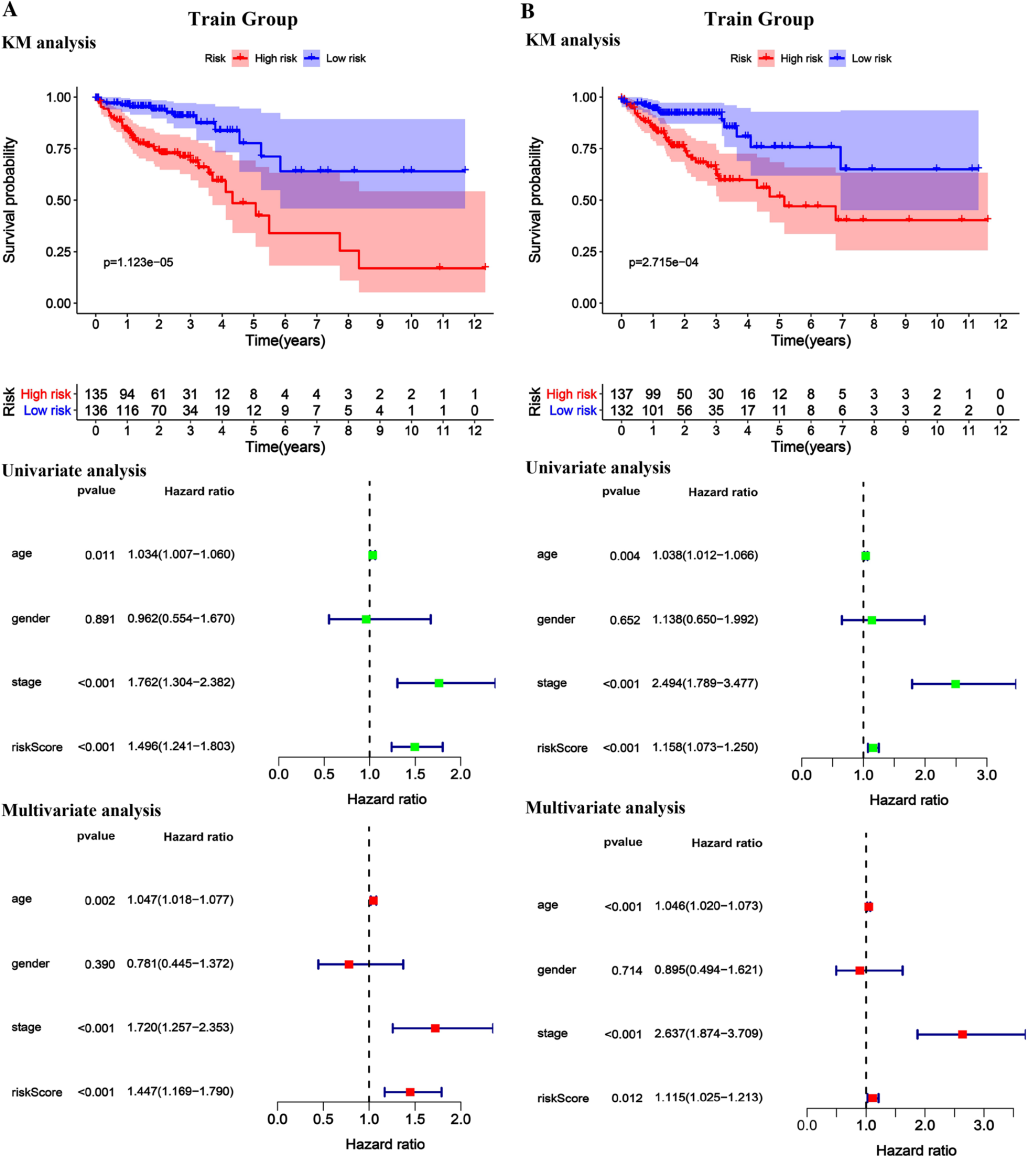
The KM survival curve, univariate independent prognostic analysis and multivariate independent prognostic analysis of the Train group (**A**) and the Test group (**B**) in the prediction model

**Fig. 7:**
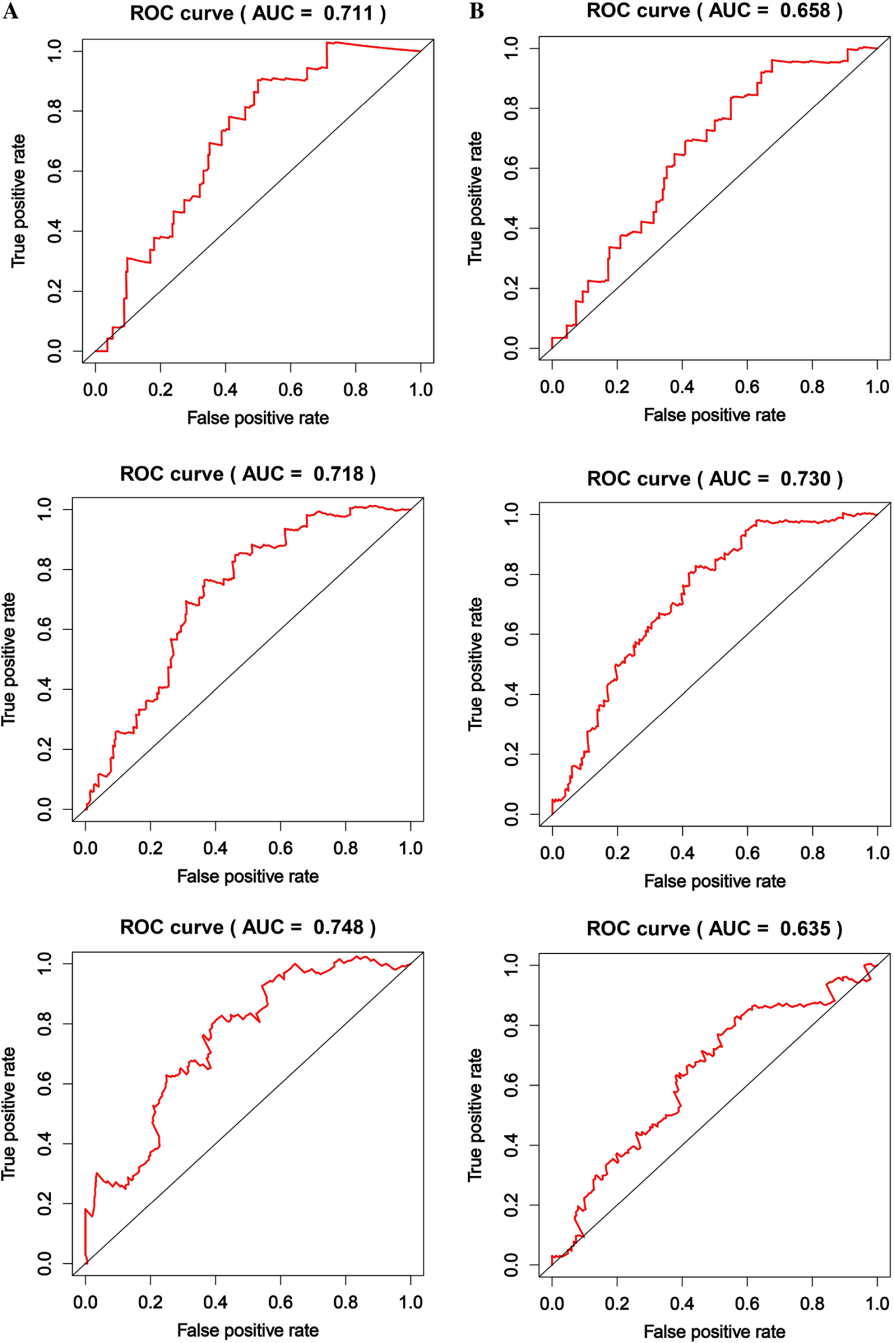
The 1-year, 3-year and 5-year ROC curves of the Train (**A**) group and Test group (**B**) in the prediction model

By using the risk score formula to combine the effects of each of these five RBP genes, the RBP risk score was calculated for each patient in the train group and the test group. According to the RBP risk score, CRC patients were divided into low-risk and high-risk groups (Fig. 5). We conducted a survival analysis on the risk scores of the train group and the test group and determined that the risk scores were both poor prognostic indicators (Fig. 6). For further verification, univariate and multivariate independent prognostic analysis involving age, gender, and stage in the train group and the test group determined that the RBP risk score was an independent predictor of patient survival (Fig. 6). Lastly, the ROC curves of the train group and the test group were drawn using R. According to its area under the curve (AUC) value, the accuracy of the model was basically at a medium level (Fig. 7).

### Genetic alterations of five RBP signatures in CRC

**Fig. 8:**
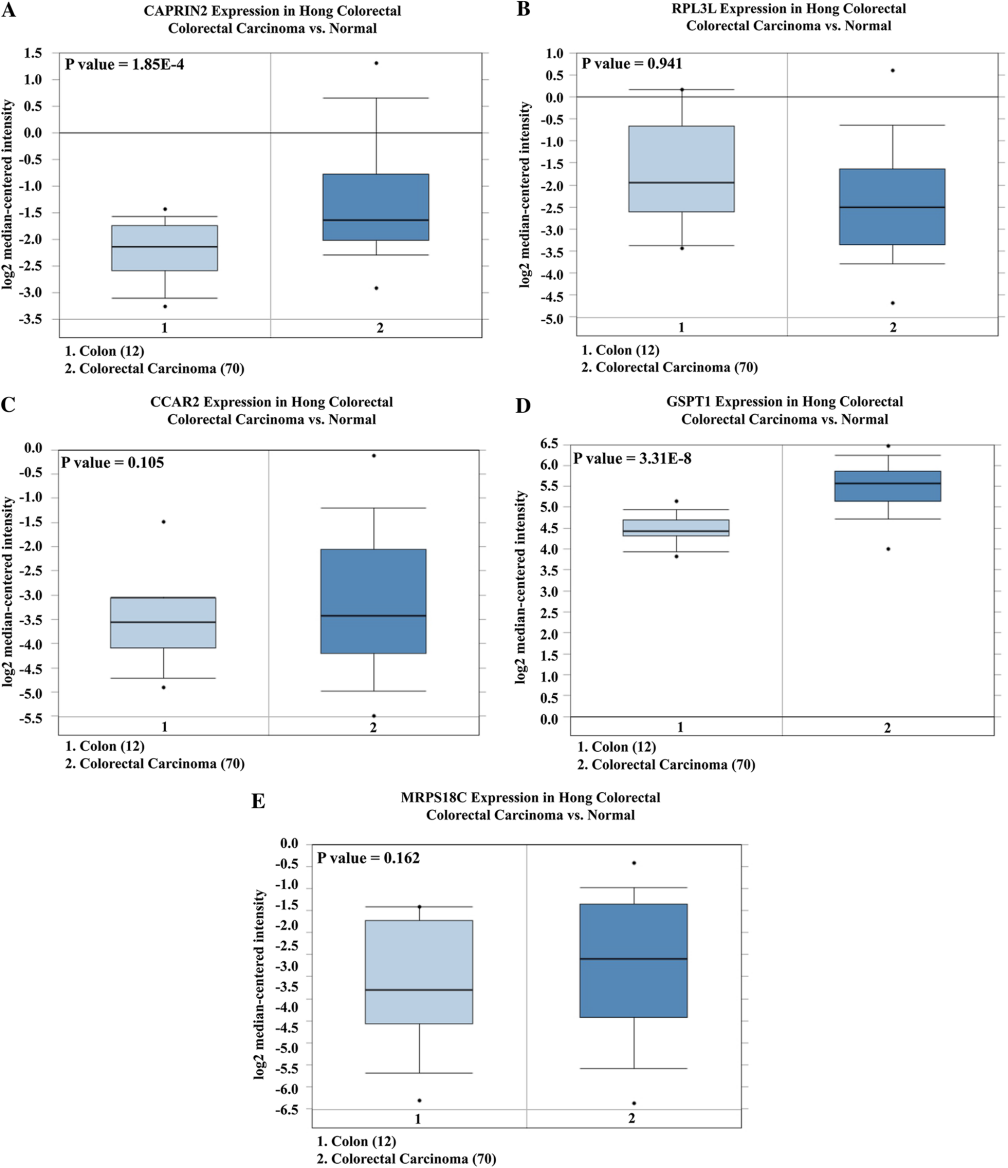
The expression of RBP genes in prognostic signatures for oncomine platform (**A**–**E**)

**Fig. 9:**
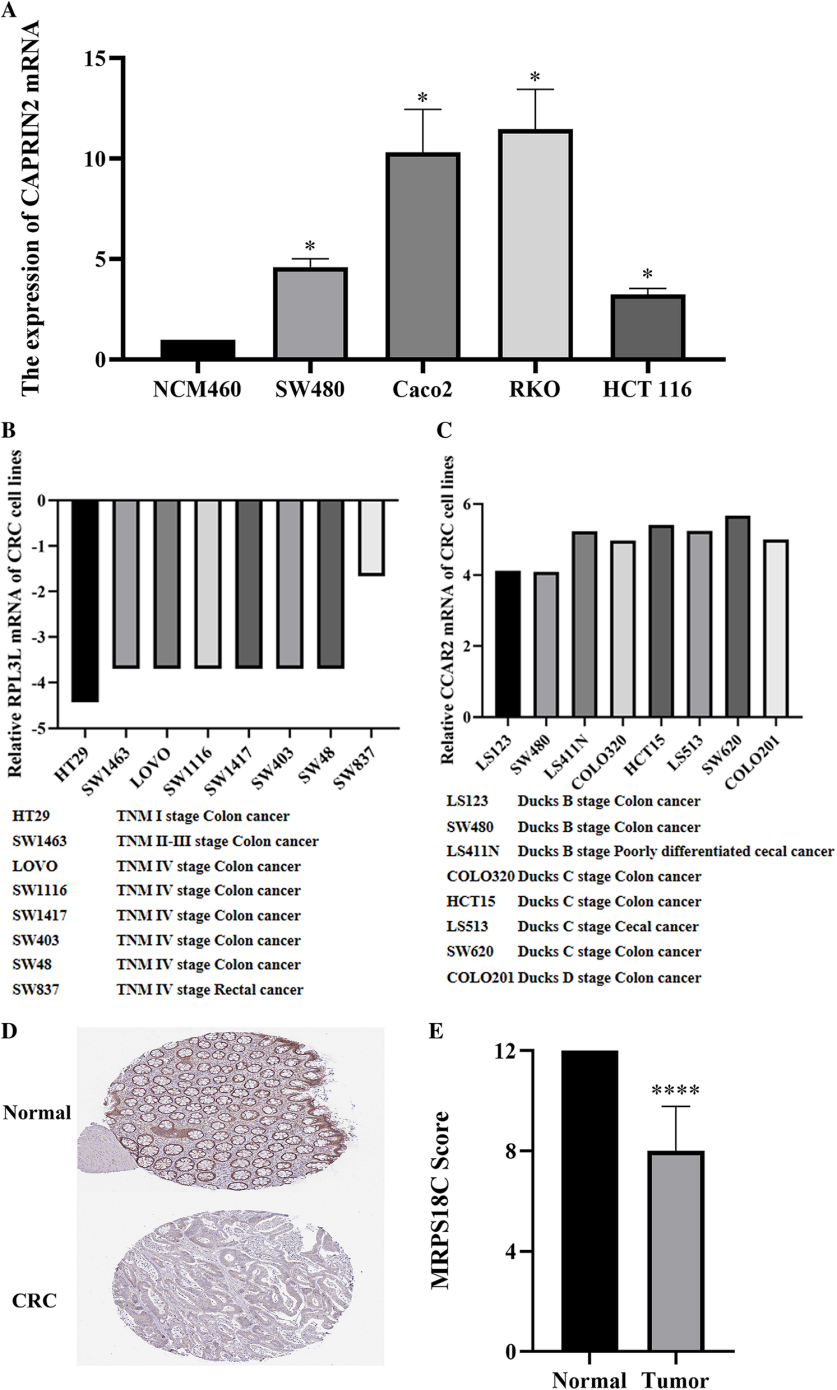
Verification of CAPRIN2 expression in CRC at RNA and protein levels (**A**). Relative expression of RPL3L mRNA in CRC cell lines from different TNM stages (**B**). Relative expression of CCAR2 mRNA in CRC cell lines from different Ducks' stages (**C**). Immunohistochemical results of MRPS18C in normal and CRC tissues (**D**–**E**). (*p < 0.05)

We put the five RBP signatures of the prognostic model on the Oncomine platform (Hong colorectal statistics) and compared the CRC samples with the normal samples. We observed that the transcription level of CAPRIN2, GSPT1 and CCAR2 (CCAR2 is also known as DBC1) showed an upward trend, while the transcription level of RPL3L and MRPS18C decreased in CRC (Fig. 8). Subsequently, we performed RT-PCR detection of CAPRIN2 mRNA in six existing CRC cell lines and normal intestinal epithelial cells (Fig. 9A), which confirmed that CAPRIN2 was indeed highly expressed in CRC. Besides, we investigated the transcription levels of the five signatures in various CRC cell lines on the CCLE platform. The expression of RPL3L mRNA in CRC cell lines of patients with TNM stage I was lower than that of patients with TNM stage II–IV (Fig. 9B). In addition, except for the LS411N cell line (from poorly differentiated CRC patient), the expression of CCAR2 mRNA in CRC cell lines of patients with Ducks' type B was significantly lower than that of patients with Ducks' type C-D, indicating that CCAR2 may be related to the progression and malignant degree of CRC (Fig. 9C). Finally, we analyzed the immunohistochemical results of five signatures in normal colorectal tissue and CRC on the platform of The Human Protein Atlas (https://www.proteinatlas.org/). According to the results of immunohistochemistry, the expression level of MRPS18C in CRC was lower than that in normal tissues (Fig. 9D and E).

## Discussion and conclusion

RBPs are a general term for a class of proteins that accompany RNA to regulate metabolic processes and bind to RNA [11]. Their main role is to mediate the maturation, transport, localization and translation of RNA. One RBP may have multiple target RNAs, and its expression defects can cause multiple diseases [10, 12]. Some scholars believed that RBP is the key to regulate the malignant transformation of CRC [20]. This study provided a comprehensive picture of RBP regulation and its importance in the occurrence and progression of CRC. In our analysis, we revealed that the main proportion of RBP in CRC samples was up-regulated. This was consistent with previously published literature, which showed that most RBPs are up-regulated in various cancers compared to their normal counterparts [20]. This suggests that most of them may have tumor-promoting effects.

After understanding the knowledge that abnormal changes in RBP may lead to CRC, we investigated the main functional pathways of abnormal expression of RBP in CRC. The enriched spliceosome pathway had attracted our attention. Genomic research showed that more than 90% of the genes in the human body have AS events [21]. This process is strictly regulated in different tissues and different physiological stages, and its imbalance leads to a variety of diseases [14]. The in vivo regulation of AS is mainly achieved by the recruitment of trans-acting splicing factors by cis-elements in the precursor mRNA [13]. Generally, trans-splicing factors have a modular structure, which includes one or more RNA binding domains and different functional modules [13]. However, the current research on these functional domains is still limited to a few typical splicing factors, such as the serine/arginine protein family and heterogeneous nuclear ribonucleoproteins protein family, but little is known about the functional modules of other RBPs [22]. In-depth understanding of these functional modules will provide a basis for scientists to further study and even synthesize new RNA splicing factors de novo. After realizing the importance of RBP in AS, our research constructed its network for regulating AS. We observed that RBP DDX39B could regulate multiple prognostic-related AS events and even control AS events related to different prognosis of the same genes, such as EIF2B1, EXOSC10, which showed that DDX39B may be an essential RBP affecting CRC. *DDX39B* gene encodes a member of the DEAD-box family of RNA-dependent ATPases, which mediates ATP hydrolysis during pre-mRNA splicing [23]. DDX39B protein is an essential splicing factor required for the association of U2 small ribonucleoprotein with pre-mRNA and also functions in the export of mRNA from the nucleus to the cytoplasm [23]. Tumors related to DDX39B include prostate cancer and melanoma [24, 25], and the situation in CRC has not been reported in the literature.

Cancer is the result of the interaction between environmental factors and cell genetic material, the result of multi-factor, multi-stage, and multi-gene effects, and the result of accumulation of gene mutations [26]. Therefore, cancer is a genetic disease. In the numerous human genes, proto-oncogenes and tumor suppressor genes are closely related to the occurrence and development of cancer [27]. Mutations in proto-oncogenes and tumor suppressor genes can cause cell canceration [28]. In our research, we found that these aberrantly expressed RBPs had mutations in more or less CRC samples. Among them, the two RBPs with the highest mutation frequency, PRKDC and HELZ2, were searched on the cBioportal platform. The *PRKDC* gene encodes the catalytic subunit of DNA-dependent protein kinase (DNA-PK) [29]. Together with Ku70/Ku80 heterodimeric protein, PRKDC has an effect on DNA double-strand break repair and recombination [30]. PRKDC-related pathways include AKT signaling pathway [31], and PRKDC expression has been shown to be positively correlated with the poor prognosis of CRC, which is a key factor in promoting drug resistance and proliferation of CRC [32, 33].

Epigenetics means that the DNA sequence does not change, but the gene expression has undergone heritable changes [34]. One of the epigenetic factors that we studied here was RBP methylation. Changes in gene methylation may have critical effects on gene expression. As a recognized fact, in most cases, hypermethylation of gene promoters leads to the inhibition of transcription, and vice versa [35]. In our research, we found that methylation participated in regulating the expression of RBP in CRC. It was shown that 21 abnormally expressed RBPs had hypomethylation and/or hypermethylation in CRC. Additionally, we also identified 10 RBPs with methylation sites that were related to prognosis. This may emphasize the importance of abnormally expressed RBPs in CRC, so the abnormal expression of these RBPs in CRC samples was regulated by one or more mechanisms.

Further, we identified five RBPs with prognostic significance in CRC tumor patients. These signatures composed of CAPRIN2, RPL3L, CCAR2, GSPT1 and MRPS18C divided CRC patients into low-risk and high-risk group. The five RBP signatures used to construct the model were closely related to the patient's prognosis in univariate and multivariate analysis and were independent factors of the patient's prognosis. Some studies suggest that CAPRIN2 has roles in the inhibition of cell growth, differentiation, the enhancement of classical WNT signaling and the maintenance of dendritic structure [36]. CAPRIN2 is considered to be an oncoprotein in hepatoblastoma [37] and can induce the development of oral squamous cell carcinoma via activating the WNT/β-catenin signaling pathway [38]. In the present research, we displayed for the first time that CAPRIN2 was significantly upregulated in CRC cells, which was consistent with the results of the tissue level on the online dataset. Moreover, high CAPRIN2 was remarkably associated with CRC patient survival. These findings confirmed that CAPRIN2 serves as an oncoprotein in CRC and is a candidate mRNA vaccine for CRC. RPL3L, which has similar sequence with ribosomal protein L3, is mainly expressed in skeletal muscle and heart, harming the growth of muscle tubes and affecting atrial fibrillation [39–41]. As we have seen yet, this study is the first to report the potential functional significance of RPL3L in tumor (including CRC). Here, we discovered that high expression of RPL3L as a malignant protein was positively correlated with survival time of CRC patients and elevated in CRC cell lines from patients with high TNM stage. Previous studies have reported that CCAR2 exerts as a regulative factor in cancer progression, such as breast cancer [42], gastric cancer [43], osteosarcoma [44], and hepatocellular carcinoma [45]. Notably, a study found that CCAR2 maintains the stability of p53 in the nucleus, promoting p53 to exert its tumor suppressor transcription function [46]. In CRC, CCAR2 enhances the cell growth and tumorigenic potential and positively regulates the WNT signaling pathway [47].Our study also observed that the level of CCAR2 increased in CRC cell lines from patients with higher Ducks' stage. These results indicated CCAR2 is promising target. GSPT1 is involved in the regulation of mammalian cell growth [48]. Long non-coding RNAs interact with microRNAs to indirectly regulate the target gene GSPT1 to mediate cellular proliferation, migration and invasion in glioma [49], cervical cancer [50], and non-small cell lung cancer [51]. It is also highly expressed in HCT 116, one of CRC cell lines [52], which is consistent with the result of our analysis at the tissue level. MRPS18C, namely mitochondrial ribosomal protein S18C, is encoded by nuclear genes and contributes in protein synthesis within the mitochondrion [53]. But at present, there is no research on MRPS18C in tumor. Here, MRPS18C was identified as a protective protein according to univariate cox regression analysis and immunohistochemistry analysis. In brief, these results imply that the above five RBPs might be involved in occurrence and development of CRC, but whether they affect the biological function of CRC cells, such as cell proliferation, still need to be further explored.

The clinical characteristics of CRC, such as pathological type, tissue type, and location of occurrence, are significantly different in different patients, and the prognosis of patients with the same stage is also different [54]. Therefore, more understanding of the prognostic factors of CRC is needed. The model divided patients into high-risk groups and low-risk groups. The survival rates between the two groups were significantly different. The 1-year, 3-year, and 5-year ROC curve AUC drawn by the train group were all greater than 0.70. The AUC of 1-year, 3-year and 5-year ROC area of test group were greater than 0.63, which further verified the model. Collectively, this prediction model may be used as a useful supplement to TNM stage.

To the best of our knowledge, this study provided the first relatively comprehensive view of the abnormally regulated RBP in CRC and its mechanism, which may result in abnormal regulation. It also provided insights into the regulation of RBP related to AS events and insights that may be related to CRC-associated pathways. We also developed the RBP signatures, which proved to be reliable independent prognostic factors in CRC. This may be clinically helpful, while making treatment-related decisions for CRC patients. However, as an exploratory study, its application value still needs to be further verified by multi-center large sample clinical research.

## Supplementary Information

**Additional file 1**. The summary of RBPs and their details.

**Additional file 2**. The abnormally expressed RBPs and RBPs related to CRC survival.

**Additional file 3**. The AS events related to CRC survival.

**Additional file 4**. The gene mutation data of 8930 CRC samples from the COSMIC website.

**Additional file 5**. The TCGA 450k array downloaded from UCSC and RBPs with prognostic methylation sites.

**Additional file 6**. The expression levels and corresponding methylation levels of interested RBP genes from the CCLE platform.

**Additional file 7**. The clinical information and relative expression levels of RBP genes in train group and test group of CRC patients.

**Additional file 8**. The expression data of five RBP signatures in various CRC cell lines from the CCLE platform.

**Additional file 9**. The detection of CAPRIN2 expression data in NCM460, SW480, Caco2, RKO and HCT116 by RT-qPCR.

## Data Availability

All data and materials could be found in our published paper.

## Abbreviations

RBP: RNA binding protein
CRC: Colorectal cancer
AS: Alternative splicing
TCGA: The Cancer Genome of Atlas
GEPIA 2: Gene Expression Profiling Interactive Analysis 2
GO: Gene ontology
KEGG: Kyoto Encyclopedia of Genes and Genomes
PSI: Percent-spliced-in
CCLE: Cancer Cell Line Encyclopedia
KM: Kaplan–Meier
ROC: Receiver operator characteristic
AUC: Area under the curve
DNA-PK: DNA-dependent protein kinase
COSMIC: Catalogue of Somatic Mutations in Cancer
